# Multisensory Stimulation and Priming (MuSSAP) in 4-10 Months Old Infants with a Unilateral Brain Lesion: A Pilot Randomised Clinical Trial

**DOI:** 10.1155/2023/8128407

**Published:** 2023-01-06

**Authors:** Anke P. M. Verhaegh, Brenda E. Groen, Pauline B. M. Aarts, Raymond van Ee, Michèl A. A. P. Willemsen, Marijtje L. A. Jongsma, Maria W. G. Nijhuis-van der Sanden

**Affiliations:** ^1^Department of Pediatric Rehabilitation, Sint Maartenskliniek, Nijmegen, Netherlands; ^2^Research Institute for Health Sciences, IQ healthcare, Radboud university medical center, Nijmegen, Netherlands; ^3^Department of Research and Innovation, Sint Maartenskliniek, Nijmegen, Netherlands; ^4^Department of Rehabilitation, Donders Institute for Brain, Cognition and Behavior, Radboud university medical center, Nijmegen, Netherlands; ^5^Department of BioPhysics, Donders Institute for Brain, Cognition and Behavior, Radboud University, Nijmegen, Netherlands; ^6^Department of Pediatric Neurology, Amalia Children's Hospital, Radboud university medical center, Nijmegen, Netherlands; ^7^Donders Institute for Brain, Cognition and Behavior, Radboud university medical center, Nijmegen, Netherlands; ^8^Behavioural Science Institute, Radboud University Nijmegen, Netherlands

## Abstract

**Aim:**

To explore the effect of an Early Intensive-Upper Limb intervention (EI-UL) compared to EI-UL with integrated Multisensory Stimulation And Priming (MuSSAP) training on improving manual ability in infants with a unilateral brain lesion.

**Method:**

A pilot randomised clinical trial with pre- and postintervention and follow-up measurements (T0, T1, and T2) was conducted. Sixteen infants with a unilateral brain lesion (corrected age is 4-10 months) received home-based intervention with video coaching. Eight infants received EI-UL and eight infants received EI-UL with integrated MuSSAP training. Primary outcome was the Hand Assessment for Infants (HAI) score. Additionally, effects were explored on initiation of goal-directed movements in both groups and on attention in the EI-UL with integrated MuSSAP training group.

**Results:**

No significant group differences in HAI scores were found. Overall, HAI ‘Affected hand score' increased between T0 and T1 (*p* = 0.001, Cohen's d = 1.04) and between T0 and T2 (*p* < 0.001, Cohen's d = 1.28); and the HAI ‘Both Hands Measure' increased between T0 and T1 (*p* < 0.001, Cohen's d = 1.72) and between T0 and T2 (*p* < 0.001, Cohen's d = 1.81). At the start of the intervention, six infants (three in both groups) did not demonstrate initiation of goal-directed contralesional upper limb movements. During the intervention one infant receiving EI-UL and all three infants receiving EI-UL with integrated MuSSAP training started to initiate goal-directed movements.

**Conclusion:**

The results suggest manual ability of infants with unilateral brain lesion improved with both interventions. We hypothesize that the integrated MuSSAP training may facilitate attention and initiation of contralesional upper limb goal-directed movements. This trial is registered with NCT05533476).

## 1. Introduction

Limited hand function in children with unilateral cerebral palsy (uCP) reduces their opportunities to participate in daily life activities and might, in turn, affect their quality of life [1]. About 50-90% of the children with uCP seem to make insufficient use of the residual functions of the affected upper limb, a phenomenon referred to as developmental disregard (DD) or learned nonuse [2, 3]. DD has been associated with neglect like symptoms resulting in spatial attention disregard on the affected side [4, 5]. In addition, DD is considered as a developmental delay due to a lack of use of the affected upper limb during sensitive developmental periods [5]. Therefore, early intervention including training of attention to the contralesional space seems to be needed to target DD [4, 5].

International clinical practice guidelines recommend early intervention with intensive task-specific training and parental coaching [6, 7]. There is limited, but promising evidence for such early intensive upper limb (EI-UL) home-based training programs [8–10]. Although previously suggested [4, 5], to our knowledge, no early training program included training of attention for the affected upper limb to reduce DD.

We, therefore, developed a novel tool and training aiming to improve attention towards the contralesional upper limb: Multisensory Stimulation and Priming (MuSSAP). Repeatedly applying rhythmically synchronous multisensory stimulation has been proposed to be helpful in provoking attention and to facilitate readiness to initiate a goal-directed movement (i.e., priming effect) at the stimulated side [11]. To train attention for the affected upper limb, we used a custom-made wristband, that provided simultaneously rhythmic visual, auditory, and tactile input. This MuSSAP training was integrated into an EI-UL home-based intervention with a video coaching approach.

The primary aim of this pilot Randomised Controlled Trial (RCT) was to explore the short-term effect of EI-UL compared to EI-UL with integrated MuSSAP training (EI-UL–MuSSAP) on the improvement of manual ability in infants with a unilateral brain lesion. In addition, we aimed to explore the individual developmental trajectories regarding self-initiated goal-directed movements in both groups and attention in the EI-UL–MuSSAP training group, and to evaluate the feasibility of the training.

## 2. Methods

### 2.1. Design

A pilot RCT with pre- and postintervention, and follow-up (at eight weeks postintervention) measurements was undertaken to explore the short-term effects of an eight-week EI-UL intervention compared to an eight-week EI-UL–MuSSAP intervention in infants with a unilateral brain lesion, both in a home-based setting with a video coaching approach. The study was reported in accordance with the CONSORT Statement (checklist in Appendix 1) and conducted in accordance with the Declaration of Helsinki at the Sint Maartenskliniek, the Netherlands from 2016–2018. The study was approved by the local ethics committee of the Region Arnhem-Nijmegen (protocol reference number: 2015-1661; NL52823.091.15).

### 2.2. Participants

In this pilot RCT study we aimed to include sixteen infants with a unilateral brain lesion. Additionally, as the norm reference scores for the Hand Assessment for Infants (HAI) [12] were not yet available at the time of our study, we also collected HAI data of typically developing infants as a reference.

Infants with a (corrected) age between 4 and 10 months at risk for uCP were recruited from eight hospitals across the Netherlands. Eligibility criteria were a diagnosis of unilateral acquired brain abnormalities (parenchymal haemorrhage or middle cerebral artery stroke) i.e. should be at risk of uCP, based on at least one cerebral imaging study (ultrasound or MRI). Exclusion criteria were severe epilepsy, severe sensory deficits (blindness, deafness), bilateral involvement, or both parents being unable to communicate in Dutch, English, or German.

Parents of eligible infants received an information letter about the study from their medical doctor. If interested, parents were invited for a visit to the rehabilitation center. During the visit an initial observation of the infant's motor performance was conducted by a paediatric rehabilitation physician and an occupational therapist. Parents received additional oral and written information about the intervention and detailed information about the study procedure. All parents of the participating infants signed an informed consent prior to participation. Infants were randomised to the intervention groups by using block randomisation. Every first infant referred by a paediatric neurologist from one of the hospitals was allocated either to EI-UL or EI-UL–MuSSAP by drawing lots (without replacement) by an independent person. Every second infant referred by the same hospital was automatically allocated to the other intervention group.

The typically developing infants were age-matched to the infants in the MuSSAP group and recruited from a child daycare centre. These infants had to be born at term and have a normal birth weight as reported by the parents.

### 2.3. Interventions

#### 2.3.1. Early Intensive-Upper Limb Intervention (EI-UL)

**Figure 1:**
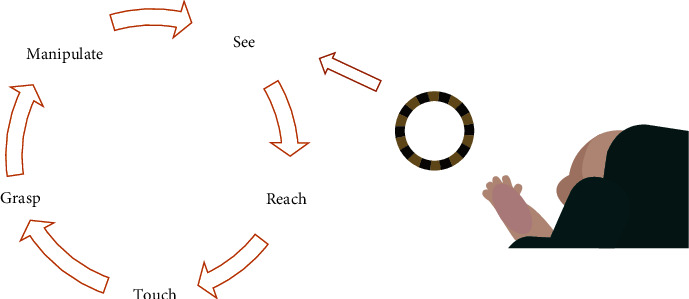
Early Intensive-Upper Limb (EI-UL) intervention. Stimulating active, self-initiated goal-directed upper limb movement by variable practice with increasing task difficulty aimed to improve performance through repeated motor activity and related sensory feedback (based on Corbetta & Snapp-Childs, [13]).

The EI-UL intervention was designed as an intensive home-based intervention with video coaching of the parents. The training consists of unimanual training (nonconstraining) stimulating child-initiated movements (like reaching, grasping, holding, and releasing) to improve contralesional upper limb ability by increasing sensory-motor experiences [13] (see Figure 1). In addition, bimanual behaviour (e.g., bimanual holding of toys) of the infant was stimulated. Individual training goals were set by the parents together with the occupational therapist. Variable practice was applied with increasing task difficulty at just the right level for each individual infant (varied toy properties or positions) [14].

The intervention consisted of eight weeks training with a frequency of seven days a week and a duration of 30 minutes each day (divided over three 10-minute sessions). Usual care (mostly paediatric physical therapy) continued during the intervention.

During a home visit at the start, the occupational therapist (>10 years of experience in working with children with CP) of the Sint Maartenskliniek provided detailed oral and written instructions to parents. The paediatric physical therapist was invited to join the parent instruction. In addition, parents received a box filled with carefully selected age and ability-appropriate toys to elicit both unimanual and bimanual actions and a stand for their telephone to be able to record the training sessions.

During the EI-UL intervention, the occupational therapist's role shifted from trainer to coach by means of video coaching. Parents were asked to record one of the 10-minute sessions every day and to upload this video on a secure website. An occupational therapist of the Sint Maartenskliniek provided parents with written feedback, training suggestions, and emotional support after analysing the videos. Parents received feedback on subsequent steps in motor skill development, how to match the difficulty of the task to the infants' ability level, how to keep practice fun, how to choose the right toys etc. The frequency of the video coaching differed (with a minimum of twice a week) and was led by the needs of the parents and observations of the therapist about treatment fidelity. Once a week, the primary care paediatric physical therapist (PT) visited parents to support parents at home during a training session.

#### 2.3.2. Multisensory Stimulation and Priming (MuSSAP) Training

**Figure 2:**
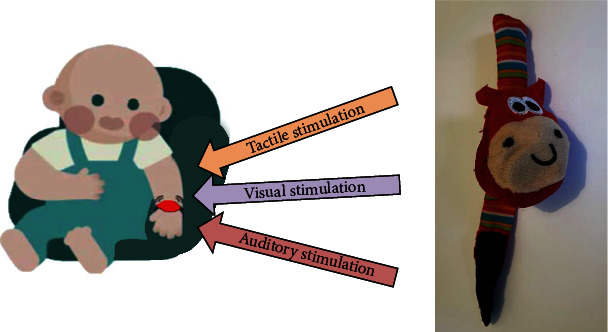
MuSSAP training. In the MuSSAP training, the infant wears a multisensory stimulating wristband (with simultaneous rhythmic visual (led lights), auditory (song), and tactile (vibration) stimuli) to increase attention for the contralesional upper limb. The multisensory wristband worn by the infants in the study is presented in the photo on the right side.

The MuSSAP training was integrated into the EI-UL intervention; the total training frequency and duration was equal to the EI-UL intervention alone. With the integrated MuSSAP training, we aimed to increase infant's attention for the contralesional upper limb and facilitate readiness to initiate a goal-directed movement [4, 11] by presenting the infant with simultaneous rhythmic visual (led lights), auditory (song), and tactile (vibration) input by a custom-made wristband attached to the contralesional wrist (see Figure 2). Based on previous research by Van Ee et al. [11], the wristband was programmed with a frequency of one stimulus per second for 20 seconds followed by a pause with a random duration of 20-40 seconds to avoid habituation to the stimulus. The multisensory input was repeated for 10 minutes before the wristband switched off automatically.

Parents closely observed whether the infant looked at the wristband after it switched on and presented a toy to the infant as soon as the infant shifted its attention to the contralesional upper limb. The toy served as a reward for shifting attention to the wristband, and as a cue to provoke self-initiated goal-directed movements with the contralesional upper limb. In case no shift of attention by the infant was observed, parents prompted attention by bringing the contralesional upper limb into the infants' field of view. No toy was presented if the infant still did not look at the wristband. In case the infant shifted its attention to the contralesional upper limb, but no self-initiated movement was observed, parents placed a toy into infant's contralesional hand to stimulate holding. The infant was left to play with the toy for as long as the contralesional hand was involved and the infant payed attention to the contralesional upper limb (in unimanual or bimanual play). If the infant transferred the toy to its ipsilesional hand or the parent observed only mouthing behaviour, the parent removed the toy. Subsequently, parents started playing peek-a-boo, talking, or singing a song prompting the infant to shift its attention to the parent until the next stimulus by the wristband occurred. In addition to the instructions for the EI-UL intervention as described earlier, parents received a supplemental instructional video demonstrating the specific MuSSAP training procedure.

### 2.4. Measurements

Infants were evaluated just before the start of the intervention (T0), immediately after the eight-week intervention (T1), and at follow-up, eight weeks after the intervention stopped (T2). All infants at risk of uCP were evaluated with the primary outcome measure; the Hand Assessment for Infants (HAI [15, 16]), and with the secondary outcome measures: the Bayley Scales of Infant and Toddler Development (Bayley-III-NL [17]), the Gross Motor Function Measure (GMFM-88 [18, 19]), the Infant and Toddler Quality Of Life questionnaire (ITQOL-SF47 [20]), and the Video Observation Attention Affected Hand (VOAAH). In addition feasibility was evaluated by adherence and short closing interviews. The typically developing infants were evaluated with all outcome measures except for the VOAAH and evaluation of feasibility.

#### 2.4.1. Primary Outcome Measure: Hand Assessment for Infants (HAI)

The HAI is a criterion and norm-referenced valid and reliable tool to assess manual ability of each hand separately as well as both hands together in infants aged 3-12 months (corrected age) at risk of uCP [15, 16].

The Each Hand Sum Score (EaHS) is a score for the left and right hand separately (range 0-24), and the Both Hands Measure (BoHM) is the overall logit-based outcome measure of the HAI (range 0-100) [16]. For the EaHS a change of ≥2 points and for the BoHM a change of ≥3 HAI units can be considered a true change [15].

As there was only one trained and certified HAI assessor (AV) in the Sint Maartenskliniek, blinded scoring was not possible. To control for expectation bias, five randomly selected videos were scored by a second trained and certified HAI assessor (TS) from a different rehabilitation clinic in the Netherlands. Outcomes were discussed until consensus was reached.

#### 2.4.2. Secondary Outcome Measures

*(1) General Gross and Fine Motor Measures*. The Bayley Scales of Infant and Toddler Development–Third Edition (Dutch version) (Bayley-III-NL) [17] is a norm-referenced, valid, and reliable instrument to assess developmental functioning of infants and young children [21, 22]. The Bayley-III is used to identify children with developmental delay. The gross motor (GM) subscale raw scores (range 0-72) were determined on all assessment points, the fine motor (FM) subscale raw scores (range 0-66) on T0 only.

The Gross Motor Function Measure (GMFM-88) is a reliable and valid multidimensional instrument to assess change in gross motor abilities in children with CP [18, 19]. The total score (i.e., average of percentage scores across five dimensions) was determined on all assessment points.

*(2) Infant and Toddler Quality of Life Questionnaire Short Form 47*. The Infant and Toddler Quality Of Life questionnaire Short Form 47 (ITQOL-SF47, Dutch version) is a reliable and valid generic parent-completed measure to assess health status and health-related quality of life of children between two months and five years old [20]. Raw scale scores are transformed to a standardized 0-100 continuum with a higher score reflecting a better health status [20].

*(3) Video Observation Attention Affected Hand (VOAAH)*. To explore the individual developmental trajectories regarding self-initiated goal-directed movements in both groups and attention in the EI-UL–MuSSAP training group, we developed the Video Observation Attention Affected Hand (VOAAH).

To assess *time to self-initiated goal-directed movement* and *time to attention,* training session videos recorded by parents were scored. *Time to self-initiated goal-directed movement* was defined as the time between looking at the stimulus (a toy presented by the parent) and initiation of a goal-directed movement (active movement towards the toy) with the contralesional upper limb. In addition, *time to attention* for the contralesional upper limb was defined as the time between the stimulus (multisensory stimulation by the wristband) and looking at the contralesional upper limb. This latter could only be scored from the videos of the infants who received EI-UL–MuSSAP.

We aimed to score two videos of each intervention week with a minimum of two days in between (in total 16 videos per infant). Videos or trials were excluded if (1) the arms or eyes of the infant could not be observed due to a wrong camera position; (2) the quality of the video was too low to be scored properly; (3) the wristband was switched on by the parent during the trial; (4) a deviation of the intervention protocol was observed (e.g., the parent presented a toy before the infant looked at the wristband); and (5) obvious environmental distraction by the infant was observed. Selected videos were scored in a nonchronological order to reduce expectation bias. From each video, the first five consecutive trials that met the inclusion criteria were scored.

A scoring guideline (Video Observation Attention Affected Hand (VOAAH)) was developed for this purpose. Evaluation of the scoring guideline showed excellent (ICC 0.984) and good (ICC 0.889) intrarater reliability for *time to self-initiated goal-directed movement* and *time to attention,* respectively (unpublished internal report).

*(4) Feasibility*. To evaluate the feasibility of the intervention, we measured training adherence by asking parents to fill out a digital time registration form every day. Additionally, a short closing interview was conducted with parents and the physical therapist immediately postintervention by using open ended questions regarding their experiences with the intervention. These interviews were audio-recorded.

### 2.5. Statistical Analysis

Descriptive statistics were used to present study data. The Shapiro-Wilk test was used to confirm normality of the data. To test for possible differences between groups at baseline (T0), independent sample *t*-tests were used. A mixed ANOVA was used to compare the effects of both interventions on HAI with Time (T0, T1, T2) as within factor and Intervention Group (EI-UL/EI-UL – MuSSAP) as between factor. For post hoc analyses Bonferroni correction was used to adjust for multiple comparisons. Effect sizes were calculated using Cohen's d, and were interpreted as small (d <0.5), medium (d = 0.5–0.8), or large (*d* ≥ 0.8) effects [23]. Analyses were carried out using IBM SPSS Statistics software (version 27).

The BSID-III scores and GMFM total scores were used to describe overall motor development and to visually explore differences in the developmental trajectories of infants between groups using graphs. Scores on the different scales of the ITQOL-SF47 were compared between groups. A difference of ≥10 points in mean scores was regarded as a reasonable difference based on the reported standard deviations found in previous studies [20, 24].

For both groups, intraindividual *time to self-initiated goal-directed movement* and for the EI-UL–MuSSAP group *time to attention* were explored via linear regression performed using GraphPad Prism version 6.07 for Windows, GraphPad Software, La Jolla California USA, http://www.graphpad.com. Individual best-fit regression lines were determined on the best three reaction times per video. These individual regression lines were averaged to compute regression lines per intervention group. No further statistical tests were conducted. Additionally, to explore the individual developmental trajectories regarding self-initiated goal-directed movements, further visual data exploration was used.

Adherence will be calculated as the percentage of the registrated total training time with respect to the total planned training time (28 hours). To analyse the short closing interviews with parents and the physical therapist, interviews were transcribed verbatim and summarized related to the open-ended questions regarding their overall experiences with the intervention, their perception of the outcome of the intervention, their perception of potential barriers in the execution of the training program and their perception of using the multisensory bracelet (only in the EI-UL – MuSSAP intervention group).

## 3. Results

**Figure 3:**
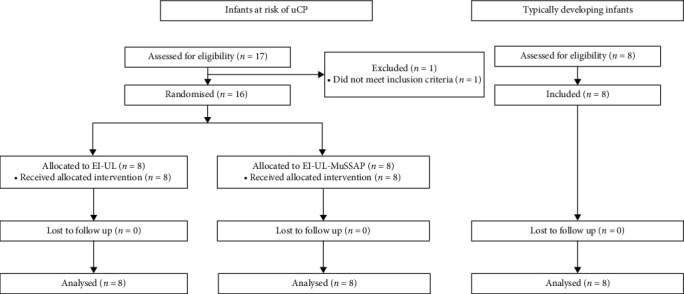
Participant flow chart.

Seventeen infants with a unilateral brain lesion were assessed for eligibility of which sixteen infants were included (Figure 3).

Unilateral brain lesion had been demonstrated by MRI (*n* = 14) or ultrasound (*n* = 2). Eight infants were randomised to the EI-UL intervention, and eight infants to the EI-UL–MuSSAP intervention. Additionally, eight typically developing infants were enrolled in the study.  Table 1 shows the demographic and baseline (T0) characteristics of the participants. At T0, no differences between intervention groups were found while in general significant differences were found between the infants with a unilateral brain lesion and the typically developing infants at T0.

Parents of three infants (two receiving EI-UL and one receiving EI-UL–MuSSAP training) reported occasional observations of a swollen, red, and warm contralesional upper limb, which was already present before the start of the intervention in two infants and in one infant from the start of the intervention. In all cases, it was recommended to consult the infants' physician, and no contraindications to proceed with the intervention were determined.

### 3.1. Primary Outcome Measure: Hand Assessment for Infants (HAI)

**Figure 4:**
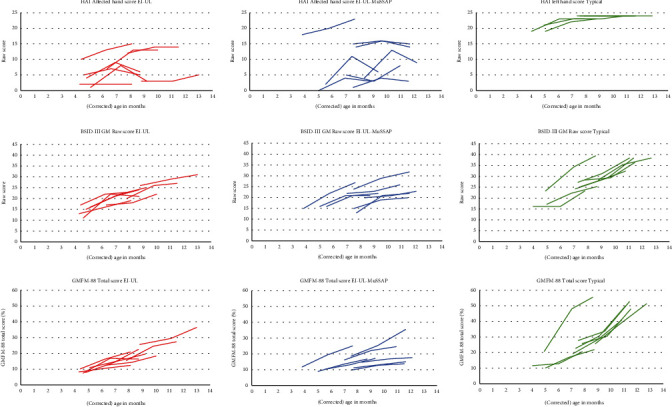
HAI affected hand scores, BSID-III GM raw scores and GMFM-88 total scores for the intervention groups and typically developing infants on T0, T1, and T2 on the *y*-axis, and (corrected) age in months on the *x*-axis.

Figure 4 shows the individual contralesional hand scores of both intervention groups and left-hand scores of typically developing infants, respectively. We observed lower scores and more intraindividual variability in the intervention groups compared to typically developing infants.

The mixed ANOVA revealed no Group and no Group × Time interaction effects for the HAI Affected Hand Score (*F* (1, 14) = 0.576, *p* = 0.460, *F*(2, 28) = 0.048, *p* = 0.953) nor for the HAI Both Hands Measure (*F*(1, 14) = 1.034, *p* = 0.327, *F*(2, 28) = 0.017, *p* = 0.983) (see Table 2). Significant time effects for the HAI Affected Hand Score (*F*(2, 28) = 12,315, *p* < 0.001) and HAI Both Hands Measure (*F*(2, 28) = 30.044, *p* < 0.001) were found. Post hoc analyses showed significant improvements in the HAI Affected Hand Score between T0 and T1 (*p* = 0.001, Cohen's d = 1.04, *Δ*3.3) and between T0 and T2 (*p* < 0.001, Cohen's d = 1.28, *Δ*2.6) and in the HAI Both Hands Measure between T0 and T1 (*p* < 0.001, Cohen's d = 1.72, *Δ*9.1) and between T0 and T2 (*p* < 0.001, Cohen's d = 1.81, *Δ*9.6). In both intervention groups six infants (75%) showed an (≥2 point raw score [15]) improvement in the Affected Hand Score between T0 and T1 and between T0 and T2. In both intervention groups three infants showed a decline in Affected Hand Score between T1 and T2. A large interindividual variability in Affected Hand Raw Scores was observed.

### 3.2. Secondary Outcome Measures

#### 3.2.1. General Gross and Fine Motor Measures

Figure 4 shows the gross motor function over time (BSID-III-NL and GMFM-88) in the intervention groups and typically developing infants. A slower increase of gross motor function over time in infants with unilateral brain lesion compared to typically developing infants was observed. Additionally, a large interindividual variability in scores between infants was seen.

#### 3.2.2. Infant and Toddler Quality of Life Questionnaire Short Form 47

In general, scores on the ITQOL were lower (≥10 points) in the intervention groups compared to scores of the typically developing infants. In case of differences in scores (defined as ≥10 points) between the intervention groups, infants in the EI-UL–MuSSAP group showed lower scores regarding quality of life compared to infants in the EI-UL group, except for the mean score on Physical Abilities on T1 (see Table 3).

In the EI-UL–MuSSAP group scores on Physical Abilities and Parental Impact-Emotional increased (≥10 points) at T1 compared to T0 and the score on the General Health Perceptions scale declined at T1 compared to T0.

#### 3.2.3. Video Observation Attention Affected Hand (VOAAH)

For 11 infants 16 videos were eligible for scoring. For two infants in both intervention groups the number of eligible video recording ranged from 10-15. For one infant (EI-UL) no video recordings were available due to video-storage failure.

**Figure 5:**
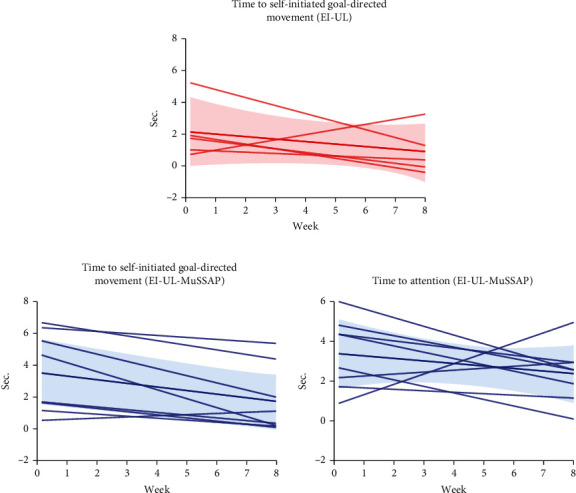
(a) Linear regression lines *time to self-initiated goal-directed movement* for infants receiving EI-UL (individual and group + SD). (b) Linear regression lines *time to self-initiated goal-directed movement* and *time to attention* for infants receiving EI-UL – MuSSAP training (individual and group + SD).

Based on visual exploration, at group level, the linear regression analysis (Figure 5) showed in both intervention groups a negative slope for both the regression lines regarding *time to self-initiated goal-directed movements* with the contralesional upper limb. In infants receiving EI-UL–MuSSAP training, the regression lines regarding *time to attention* also showed a negative slope suggesting faster response times. Infants receiving EI-UL appeared to have faster response times at T0 compared to infants receiving EI-UL–MuSSAP training and remained faster during the intervention period.

The individual developmental trajectories regarding self-initiated goal-directed movements were visually explored using the figures in Appendix 2. Of the infants receiving EI-UL, three infants did not demonstrate goal-directed movements with the contralesional upper limb in the first intervention week. One of these infants acquired goal-directed movements from the eighth intervention week onwards and two of these infants did not acquire consistent goal-directed movements during the intervention period. Of the infants receiving EI-UL–MuSSAP training, three infants did not demonstrate goal-directed movements with the contralesional upper limb and demonstrated poor reaction times regarding attention for the multisensory stimulus in the first intervention week. All these three infants acquired goal-directed movements on average from the fourth intervention week onwards.

### 3.3. Feasibility

#### 3.3.1. Adherence

The average received training duration of the EI-UL and EI-UL–MuSSAP group was similar in both groups; 93% (26 SD = 2 hrs) and 86% (24 hours SD = 6 hrs), respectively, (*p* = 0.313) and there were no dropouts. For two infants receiving EI-UL–MuSSAP adherence rate was <50% (46% and 49%, respectively) due to multiple hospital admissions and difficulties with fitting the intervention into daily life.

#### 3.3.2. Closing Interview

Most parents and paediatric physical therapists in the EI-UL intervention group were positive about the intervention, except for the parents and the therapist of one infant who experienced the intervention as very intensive. Seven parents and six therapists had observed improvements in the infants' upper limb function.

Most parents and all paediatric physical therapists involved in the EI-UL–MuSSAP group were positive about the training and had observed improvements in the infants' upper limb function. Parents told that the wristband stimulated the infant to shift its attention to the contralesional upper limb, although that could take several weeks. Some physical therapists mentioned the bracelet provided the stimulus that was needed to direct attention to the contralesional upper limb. One parent speculated it might have been possible to train without the wristband, however, it probably would have taken much longer to direct attention to the affected upper limb. Parents explained that the infant eventually connected the multisensory stimulation of the wristband with reminding of and using the contralesional upper limb. The paediatric physical therapists appreciated the highly structured format of three times 10 minutes of training, especially with the wristband being switched off automatically after 10 minutes. Two therapists mentioned the delivery of the MuSSAP training protocol was difficult and required some practice.

## 4. Discussion

This pilot randomised controlled trial showed no difference on the HAI scores for the affected hand and both hands between infants receiving EI-UL intervention and infants receiving EI-UL–MuSSAP training. The intervention with and without integrated MuSSAP training seems to be feasible, and significant improvements and large effect sizes were found, immediately after treatment and after 8 weeks follow-up. In addition, faster *time to self-initiated goal-directed movements* with the contralesional upper limb were observed in both intervention groups as well as faster *time to attention* for the infants receiving EI-UL–MuSSAP training.

On group level, no additional beneficial effects of the integrated MuSSAP (attention) training were found. The intervention effects in both intervention groups were similar and in line with previous research exploring the effects of baby-CIMT [9]. The mean improvement in hand function development after intervention found in our study was much larger than could be expected based on previous findings [25] regarding hand function development in the first year of life in infants with uCP, taking the developmental trajectories (low, moderate, high) into consideration. We suggest that restraining the ipsilesional upper limb, being the essential approach in baby-CIMT, may not be needed to improve manual ability in infants at high risk of developing uCP. We speculate that this particularly applies for the youngest infants who do not yet cross the midline with the ipsilesional upper limb. Others have also indicated that both CIMT and bimanual training can be equal effective to improve hand function in infants at high risk of developing uCP [8].

In our study, we observed a large variation in (increase in) manual ability between infants. This observed interindividual variability was larger than observed in the typically developing infants and in accordance with previous studies [8, 9]. Disease characteristics (e.g., brain lesions and comorbidities such as epilepsy) may hamper development and intervention effectiveness. Heterogeneity in disease characteristics could account for the interindividual variability in manual ability. Training dose may also influence intervention effectiveness [26]. Noteworthy, in the two infants with a low intervention adherence (<50%) (both participating in the EI-UL–MuSSAP intervention group) we did not observe an increase in manual ability. This low adherence might have influenced the effect of the intervention. Future research is necessary to further explore what intervention duration is most beneficial to achieve favourable outcomes in each individual infant.

Visual data-exploration suggested that infants with lack of goal-directed movements at the start of the intervention and with diminished attention for the contralesional upper limb might benefit from MuSSAP training. Risk for diminished attention and goal-directed movements may be related to the location and the severity of the brain damage. From adult populations it is suggested that impaired attention to the contralesional side commonly occurs following brain damage to the right posterior parietal cortex [27]. However, due to known differences in the anatomical distribution and brain reorganization between the developing and the mature brain [28], more research is needed to confirm such a relationship in infants.

By exploring the outcomes on the measures regarding gross motor development and quality of life, infants in the intervention groups generally showed lower scores and a slower developmental rate compared to typically developing infants, confirming a correct selection of high-risk infants in the intervention groups. Infants receiving EI-UL seem to have slightly higher scores on the quality of life measure compared to infants receiving EI-UL–MuSSAP training. The lower quality of life scores in the infants receiving EI-UL–MuSSAP intervention may be associated with a larger number of infants with lower physical well-being due to respiratory disease (*n* = 1), and due to epilepsy (*n* = 2) (disclosed during the study) compared to one infant with epilepsy in the EI-UL group.

Inclusion of the infants was based on outcomes of cerebral imaging studies and the clinical judgement of the paediatric neurologist. At the time of inclusion, the asymmetry index cut-off values of the HAI [29] were not yet available. Post hoc we could conclude that one child in the EI-UL–MuSSAP scored within the normal range of the HAI and could therefore have been excluded. However, exclusion of this participant would not have changed our conclusions.

A limitation of the current study is that the effects of the intervention and the (additional) effects of development while growing two (T1) and four (T2) months older cannot be distinguished. Due to ethical concerns that would raise by withholding early intervention programs to infants at high risk of developing uCP, we did not include a group of infants with unilateral brain lesion receiving no intervention. We observed a decline in manual ability in six infants with unilateral brain lesion during follow-up, while in typically developing infants only increases were observed, as expected. The decline in manual ability during follow-up suggests the presence of an intervention effect, although the size remains unknown. It may also suggest that 8 weeks of intervention were too short to give sustained improvement. Though, in most cases, manual ability at follow up was still higher than manual ability at baseline.

Another limitation was the lack of a clinical measure for attention (for the contralesional upper limb) at the start of the study. Therefore, we developed a scoring guideline (VOAAH) for scoring the videos made by the parents of the training sessions at home. The quality of these videos, however, was sometimes poor and scoring of the videos was a time-demanding procedure. For clinical purposes, a technology-based method (for example using gaze detection) may be a more feasible and reliable approach. Furthermore, it must be noted that looking at the wristband is a proxy measure of attention for the contralesional upper limb. Adding brain-based measures (e.g., electro-encephalography) to future studies may be of added value. This is a noninvasive, affordable technique that can be applied in young infants and children with CP [30, 31].

Furthermore, blinded scoring of the outcome measures was not achieved. The occupational therapist who coached the families during the intervention also performed the measurements and scoring, since she was the only certified HAI assessor available in our clinic. By scoring a random selection of HAI assessments by a second certified HAI assessor from a different rehabilitation center in the Netherlands followed by a consensus meeting, reliability of the HAI scoring was enhanced as much as possible.

## 5. Conclusions and Implications for Clinical Practice

Our pilot study indicated no significant differences between intervention groups on manual ability. Both the EI-UL and EI-UL–MuSSAP home-based intervention with video coaching may be feasible and potentially effective with regard to improving manual ability in infants at high risk of developing uCP. As it is not (yet) possible to predict what intervention and what intervention duration is most likely to induce permanent improvement of upper limb function in each individual infant, the choice of intervention should be based on clinical reasoning, feasibility, and the preferences of the family. From the results of the current pilot study, we could form the following hypothesis: infants who do not (yet) initiate goal-directed movements and who show diminished attention for the contralesional upper limb, may benefit more from an additional (multisensory) MuSSAP training than from EI-UL alone. Our hypothesis should be further investigated in a in a larger study comparing the results of an EI-UL and EI-UL–MuSSAP home-based intervention, stratifying participants by the absence of goal-directed movements of the affected upper limb at baseline. By adding brain-based measures, possible effects of the interventions on cortical reorganisation can be monitored.

## Figures and Tables

**Table 1:** Participant demographic and baseline (T0) characteristics.

	EI-UL (*n* = 8)	EI-UL-MuSSAP (*n* = 8)	Typical (*n* = 8)
(Corrected) age in months, mean (range)	5.8 (4.3-8.8)	6.5 (3.8-8.3)	6.6 (4.0-8.8)
Gender, M/F, *n*	3/5	7/1	5/3
Preterm (GA<37 wk)/term, n	2/6	3/5	0/8
Affected hemisphere left/right, *n*	3/5	4/4	n.a.
*Neuroimaging*			
PAIS/PVHI/nonspecific, *n*	5/2/1	3/5/0	n.a.
*HAI, BSID-III-NL, and GMFM scores: mean (SD)*			
HAI Both Hands Measure	44.0 (8.0)^a^	49.3 (11.3)^a^	82.5 (17.5)
HAI Affected Hand Sum Score	5.5 (3.9)^a^	7.4 (7.1)^a^	22.3 (2.3)
BSID-III-NL GM raw score	16.9 (4.6)^a^	17.6 (3.9)^a^	23.5 (4.7)
BSID-III-NL FM raw score	13.5 (3.5)^a^	16.4 (2.9)^1^	19.4 (4.9)
GMFM total score	12.3 (6.0)^a^	12.8 (3.5)^a^	20.6 (6.6)
*ITQOL-SF47 scores: mean (range)*			
Physical abilities	69 (53-92)	63 (20-93)	99 (93-100)^1^
Growth and development	85 (55-100)	76 (55-100)	93 (75-100)
Bodily pain/discomfort	88 (75-100)	70 (38-100)	83 (63-100)
Temperament and moods	82 (71-96)	72 (42-88)	81 (63-96)
General health perceptions	66 (31-83)	61 (27-89)	88 (79-93)
Parental impact-emotional	84 (69-100)	74 (44-100)	95 (88-100)
Parental impact-time	88 (67-100)	83 (42-100)	96 (83-100)
Family cohesion	91 (60-100)	84 (60-100)	91 (85-100)

Corrected age was calculated for infants born <37 weeks. Abbreviations: EI-UL: Early Intensive-Upper Limb intervention; MuSSAP: Multi Sensory Stimulation And Priming; MRI: Magnetic Resonance Imaging; PAIS: perinatal arterial ischemic stroke; PVHI: periventricular haemorrhagic infarction; HAI: Hand Assessment for Infants; BSID-III-NL: Bayley Scales of Infant and Toddler Development-Third Edition-Dutch version; GMFM: Gross Motor Function Measure-88; ITQOL; Infant and Toddler Quality Of Life questionnaire Short Form 47. ^1^*n* = 7, ^a^significantly different compared to typically developing children (*p* < 0.05).

**Table 2:** HAI scores before intervention (T0), after intervention (T1), and eight weeks after the intervention stopped (T2).

	EI-UL (*n* = 8)		EI-UL-MuSSAP (*n* = 8)	
Time	Mean (SD)	95% CI	Mean (SD)	95% CI
HAI Affected Hand Raw Score				
T0	5.5 (3.9)	2.3-8.8	7.4 (7.1)	1.41-13.34
T1^a^	8.6 (4.6)	4.8-12.4	10.9 (6.5)	5.4-16.3
T2^a^	7.9 (5.2)	3.5-12.3	10.1 (6.9)	4.4-15.9
HAI Both Hands Measure				
T0	44.0 (7.9)	37.4-50.6	49.3 (11.3)	39.8-58.7
T1^a^	53.4 (8.0)	46.7-60.1	58.1 (11.5)	48.5-67.8
T2^a^	53.6 (9.5)	45.7-61.5	58.8 (13.2)	47.7-69.8

*^a^* Significantly different compared to T0 (*p* < 0.05).

**Table 3:** Quality of life (mean and range) measured in both intervention groups and in typically developing infants at T0, T1, and T2.

	EI-UL			EI-UL - MuSSAP			Typical		
ITQOL-SF47 scale	T0 (*n* = 8)	T1 (*n* = 8)	T2 (*n* = 8)	T0 (*n* = 8)	T1 (*n* = 7)	T2 (*n* = 7)	T0 (*n* = 8)	T1 (*n* = 8)	T2 (*n* = 7)
Infant									
Physical abilities	69 (53-92)	65 (33-87)	68 (33-87)	63 (20-93)	76 (44-100)	72 (53-100)	99^∗^ (93-100)	100 (-)	100 (-)
Growth and development	85 (55-100)	88 (55-100)	87 (55-100)	76 (55-100)	79 (70-90)	78 (55-100)	93 (75-100)	94 (75-100)	97 (80-100)
Bodily pain/discomfort	88 (75-100)	84 (63-100)	86 (50-100)	70 (38-100)	66 (25-88)	66 (0-100)	83 (63-100)	80 (50-100)	82 (75-100)
Temperament and moods	82 (71-96)	84 (71-100)	84 (71-100)	72 (42-88)	76 (46-92)	67 (33-92)	81 (63-96)	84 (79-96)	83 (71-92)
General health perceptions	66 (31-83)	67 (52-81)	64 (38-88)	61 (27-89)	50 (26-85)	51 (18-92)	88 (79-93)	90 (85-100)	88 (68-96)
Parent									
Parental impact–Emotional	84 (69-100)	88 (69-100)	85 (63-100)	74 (44-100)	84 (63-100)	75 (13-100)	95 (88-100)	98 (81-100)	98 (88-100)
Parental impact–Time	88 (67-100)	91 (75-100)	90 (75-100)	83 (42-100)	87 (67-100)	86 (33-100)	96 (83-100)	97 (75-100)	96 (83-100)
Family cohesion	91 (60-100)	91 (60-100)	89 (60-100)	84 (60-100)	91 (85-100)	90 (60-100)	91 (85-100)	88 (60-100)	94 (85-100)

^∗^1 missing.

## Data Availability

The dataset generated and analyzed during the current study is not publicly available due to individual privacy. Data are however available from the corresponding author upon reasonable request and with permission of the parents of the participants.

## Additional Points

*Trial registration number*. The study protocol was retrospectively registered (ClinicalTrials.gov Identifier: NCT05533476).

## Supplementary Materials

Supplementary Materials Appendix 1: CONSORT checklist. Appendix 2: figures of individual developmental trajectories regarding time to self-initiated goal-directed movements in both intervention groups.
