# Supplementary material for: Multisensory Stimulation and Priming (MuSSAP) in 4-10 Months Old Infants with a Unilateral Brain Lesion: A Pilot Randomised Clinical Trial
Source: Occup Ther Int. 2023 Jan 6;2023:8128407. doi: 10.1155/2023/8128407 (PMC9839410; doi:10.1155/2023/8128407)
Supplement: Supplementary Materials — Appendix 1: CONSORT checklist. Appendix 2: figures of individual developmental trajectories regarding time to self-initiated goal-directed movements in both intervention groups. [file 8128407.f1.zip › Appendix 2. Individual dev trajectories of time to self initiated goal-directed movement.pdf]

Appendix 2: Individual developmental trajectories of *time to self-initiated goal-directed movement*

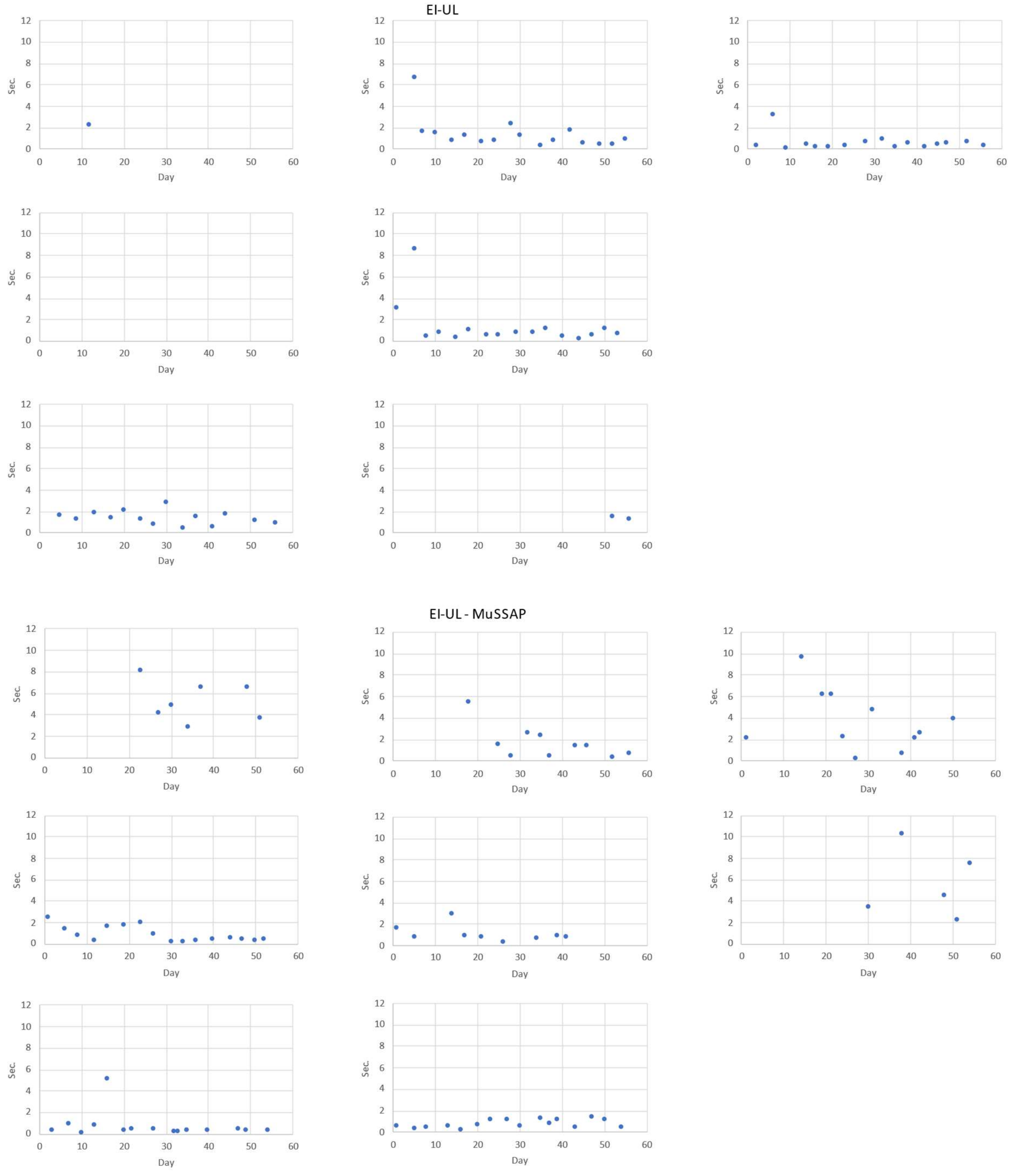

Fig Appendix 2. Individual developmental trajectories of *time to self-initiated goal-directed movement* for infants receiving EI-UL and infants receiving EI-UL – MuSSAP training
